# Targeting Elastin‐Derived Peptides Reverses Alveolar Epithelial Dysfunction in Chronic Obstructive Pulmonary Disease

**DOI:** 10.1002/mco2.70889

**Published:** 2026-07-25

**Authors:** Huijuan Zhu, Yiling Zhao, Yingchao Qin, Wenfeng Huang, Jiarui Weng, Zihan Liu, Jiahong Kuang, Zibei Feng, Zhilian Ye, Peiji Zheng, Xiaolan Guo, Fei Cui, Bingjie Chen, Pixin Ran, Jianwei Dai

**Affiliations:** ^1^ GMU‐GIBH Joint School of Life Sciences The Guangdong‐Hong Kong‐Macao Joint Laboratory For Cell Fate Regulation and Diseases State Key Laboratory of Respiratory Disease Guangzhou Medical University Guangzhou Guangdong People's Republic of China; ^2^ Department of Intensive Care Unit The Second Affiliated Hospital Guangzhou Medical University Guangzhou Guangdong People's Republic of China; ^3^ Department of Intensive Care Unit The First Affiliated Hospital Guangzhou Medical University Guangzhou Guangdong People's Republic of China; ^4^ Guangzhou National Laboratory Bio‐Island Guangzhou People's Republic of China

**Keywords:** alveolar repair, alveolar type 2 cells, chronic obstructive pulmonary disease, elastin‐derived peptides, peptide‐based therapy

## Abstract

Chronic obstructive pulmonary disease (COPD) is characterized by progressive alveolar destruction and defective regeneration, yet the matrix‐derived factors contributing to this process remain poorly defined. We investigated the role of elastin‐derived peptides (EDPs), bioactive fragments generated during cigarette smoke (CS)‐induced elastin degradation, in alveolar epithelial dysfunction. In lung tissues from patients with COPD and in CS‐exposed mice, elevated levels of EDPs were associated with elastic fiber disruption and impaired alveolar type 2 (AT2)‐to‐alveolar type 1 (AT1) differentiation. These observations were supported by histological analyses, single‐cell transcriptomic profiling, and organoid‐based functional assays. In mouse and human alveolar organoids, CS extract and EDPs each suppressed organoid growth and impaired AT2‐to‐AT1 differentiation. Mechanistically, EDPs exposure was associated with activation of TLR4/NF‐κB signaling, and reduced β‐catenin activity, whereas pharmacological inhibition of TLR4 partially restored alveolar epithelial differentiation. Notably, the EDPs‐neutralizing agent TB partially rescued AT2‐to‐AT1 differentiation in organoids and alleviated emphysematous injury in vivo. Together, these findings identify EDPs as an important matrix‐derived mediator of alveolar regenerative failure in COPD and support further evaluation of EDP‐targeted intervention as a potential strategy for promoting alveolar repair.

## Graphical abstract

In COPD, extracellular matrix and elastin degradation increase elastin‐derived peptides (EDPs), which are associated with TLR4/NF‐κB activation, DKK1 upregulation, reduced β‐catenin signaling, and impaired AT2‐to‐AT1 differentiation. Neutralization of EDPs by TB partially restores β‐catenin‐associated alveolar epithelial differentiation and attenuates alveolar injury.

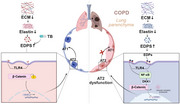

## 1 Introduction

Chronic obstructive pulmonary disease (COPD) is a progressive and life‐threatening respiratory disorder and remains one of the leading causes of death worldwide, accounting for more than 3.5 million deaths annually [1, 2]. It is characterized by persistent airflow limitation and progressive destruction of alveolar architecture, encompassing the clinical phenotypes of chronic bronchitis and emphysema [3]. Current therapies, including β2‐adrenergic agonists, anticholinergics, and glucocorticoids, primarily provide symptomatic relief and reduce exacerbation risk [4, 5]. In addition, long‐term use of some of these agents may be associated with adverse effects, including tachycardia, postural hypotension, and hyperglycemia [6, 7, 8]. Thus, elucidating the mechanisms underlying impaired alveolar repair is essential for the development of regenerative therapies for COPD.

Increasing evidence suggests that COPD is not only a chronic inflammatory disease but also a disorder of impaired tissue repair and progenitor‐cell dysfunction [9, 10, 11]. Among the epithelial cell populations involved in alveolar regeneration, alveolar type 2 (AT2) cells function as facultative progenitors and are essential for the regeneration of alveolar type 1 (AT1) cells following lung injury [12]. In the healthy lung, AT2 cells proliferate and transition through intermediate states, including damage‐associated transient progenitors (DATPs), before differentiating into mature AT1 cells to restore alveolar structure and function [13, 14, 15]. However, this regenerative process is impaired in COPD, where AT2 cells fail to complete proper differentiation, thereby contributing to defective epithelial repair and progressive alveolar destruction [16]. Although previous studies have described impaired AT2‐to‐AT1 transition in COPD and cigarette smoke (CS)‐related lung injury, the upstream matrix‐derived factors linking structural damage to progenitor‐cell dysfunction remain poorly understood [17].

A central pathological feature of COPD is the progressive destruction of the extracellular matrix (ECM), particularly elastin, which is indispensable for maintaining alveolar elasticity and structural integrity [18, 19, 20, 21]. CS exposure promotes neutrophil and macrophage infiltration and enhances the release of elastolytic enzymes, including matrix metalloproteinases and neutrophil elastase, leading to excessive elastin degradation [22, 23]. This process generates soluble elastin‐derived peptides (EDPs), which are increasingly recognized as bioactive mediators rather than inert degradation by‐products [24]. Previous studies have shown that EDPs can modulate inflammation, disrupt ECM homeostasis, and regulate the proliferation or differentiation of multiple cell types, including lymphocytes, endothelial cells, fibroblasts, and adipocytes [25, 26, 27, 28]. However, whether the accumulation of EDPs directly contributes to alveolar epithelial dysfunction in COPD, particularly impaired AT2‐to‐AT1 differentiation, remains unclear. More specifically, it is not known whether EDPs act as pathogenic mediators linking elastin degradation to defective alveolar regeneration.

These observations highlight a critical gap in our understanding of COPD pathogenesis: although ECM destruction is a defining pathological feature of the disease, the mechanisms linking elastin degradation to impaired alveolar regeneration remain incompletely understood. TB‐B002D (TB), a peptide‐based agent developed to neutralize excessive EDPs, has shown efficacy in attenuating pathological ECM remodeling in pulmonary fibrosis and has been proposed to act, at least in part, through modulation of EDP‐associated signaling and restoring ECM homeostasis [29]. Whether EDPs act as pathogenic mediators of alveolar regenerative failure in COPD and whether targeting EDP‐related signaling can preserve alveolar epithelial differentiation remain unknown.

In this study, we investigated the alterations and potential pathogenic role of EDPs in COPD, with a particular focus on their contribution to impaired AT2‐to‐AT1 differentiation and alveolar regeneration failure. We further evaluated the therapeutic potential of TB in restoring alveolar epithelial differentiation and attenuating CS‐induced COPD‐like lung injury.

## 2 Results

### 2.1 Elastin Degradation and Increased EDPs Levels in Human COPD and CS‐Exposed Mice

**FIGURE 1:**
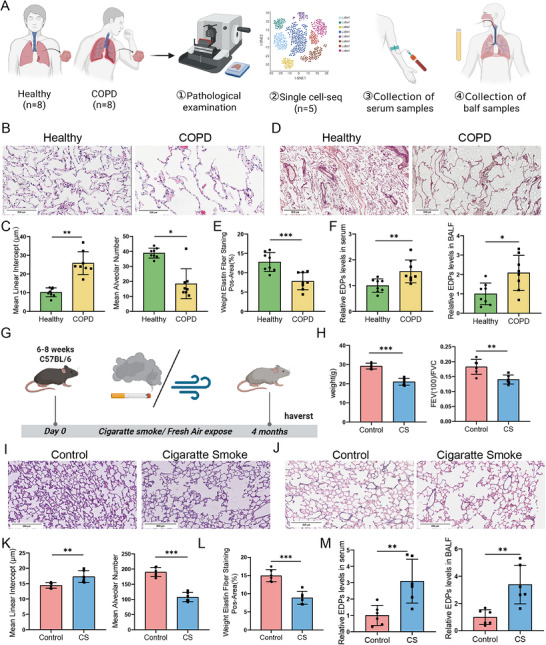
Elastin degradation and elevated EDPs levels in human COPD and cigarette smoke‐induced mice. (A) Schematic overview of the study design. Lung tissue samples were collected from healthy donors and patients with chronic obstructive pulmonary disease (COPD). In parallel, serum and bronchoalveolar lavage fluid (BALF) samples were obtained from corresponding clinical cohorts for elastin‐derived peptide (EDPs) measurement. A subset of lung tissues was subjected to single‐cell RNA sequencing (scRNA‐seq). (B, C) Representative hematoxylin and eosin (H&E)‐stained human lung sections and quantification of mean linear intercept (MLI, µm) and alveolar number per field. Scale bar, 200 µm. (D, E) Elastic fiber staining of human lung tissue and quantification of elastin‐positive area. Scale bar, 300 µm. (F) Relative EDPs levels in human serum and BALF. (G) Experimental design of the cigarette smoke (CS)‐exposed mouse model. (H) Body weight and pulmonary function, presented as forced expiratory volume in 100 ms/forced vital capacity (FEV100/FVC), after 4 months of exposure. (I, K) Representative H&E‐stained mouse lung sections and quantification of MLI (µm) and alveolar number per field. (J, L) Elastic fiber staining of mouse lung tissue and quantification of elastin‐positive area. (M) Relative EDPs levels in mouse serum and BALF. Data are presented as mean ± SEM. Human tissue/serum analyses, *n* = 8 per group; mouse analyses, *n* = 6 per group. *p < 0.05, **p < 0.01, ***p < 0.001 by unpaired two‐tailed Student's *t*‐test.

We first analyzed lung tissue samples obtained from eight age‐matched healthy donors and eight patients with moderate‐to‐severe COPD undergoing thoracic surgery (Figure 1A), with their clinical characteristics summarized in Table S1. Histopathological examination of hematoxylin and eosin (H&E)‐stained lung sections revealed characteristic emphysematous alterations in COPD lungs, including alveolar wall destruction, alveolar coalescence, and airspace enlargement. These structural abnormalities were accompanied by a significant increase in mean linear intercept (MLI) and a reduction in alveolar number per field compared with healthy controls (Figure 1B, C).

To further characterize molecular changes associated with COPD, we performed single‐cell RNA sequencing (scRNA‐seq) on a subset of human lung specimens. Differential expression analysis revealed significant upregulation of genes encoding ECM‐degrading enzymes (Figure S1), suggesting enhanced ECM remodeling in COPD lungs.

Given the critical role of elastin in maintaining alveolar architecture and lung elasticity, we next assessed elastin integrity by elastic fiber staining. COPD lung tissues exhibited pronounced elastic fiber fragmentation and disorganization (Figure 1D,E), consistent with accelerated ECM degradation. Because elastin degradation generates bioactive EDPs, we next examined whether EDP levels were altered in COPD. Notably, EDP levels were significantly elevated in both serum and bronchoalveolar lavage fluid (BALF) from patients with COPD compared with healthy controls (Figure 1F). Together, these findings demonstrate a close association between elastin degradation and increased EDP abundance in COPD.

To determine whether these alterations could be recapitulated experimentally, we established a chronic CS‐exposed mouse model of COPD (Figure 1G). Compared with air‐exposed controls, CS‐exposed mice exhibited progressive body weight loss and impaired pulmonary function, as evidenced by a significantly reduced FEV100/FVC ratio after 4 months of smoke exposure (Figure 1H). Histological analysis revealed typical emphysematous changes, including enlarged airspaces and decreased alveolar density, which were accompanied by a significant increase in MLI and a reduction in alveolar number per field (Figure 1I,K).

Consistent with the findings in human COPD tissues, elastic fiber staining revealed marked fragmentation and disorganization of elastic fibers in CS‐exposed lungs, together with a significant reduction in elastin‐positive area (Figure 1J,L). Furthermore, EDP levels were significantly elevated in both serum and BALF from CS‐exposed mice relative to air controls (Figure 1M).

Collectively, these findings identify elastin degradation and EDP accumulation as shared pathological features of human COPD and CS‐induced emphysema, supporting a potential link between ECM destruction and alveolar epithelial dysfunction.

### 2.2 Elevated Levels of EDPs Correlate With Impaired AT2‐to‐AT1 Differentiation in COPD

**FIGURE 2:**
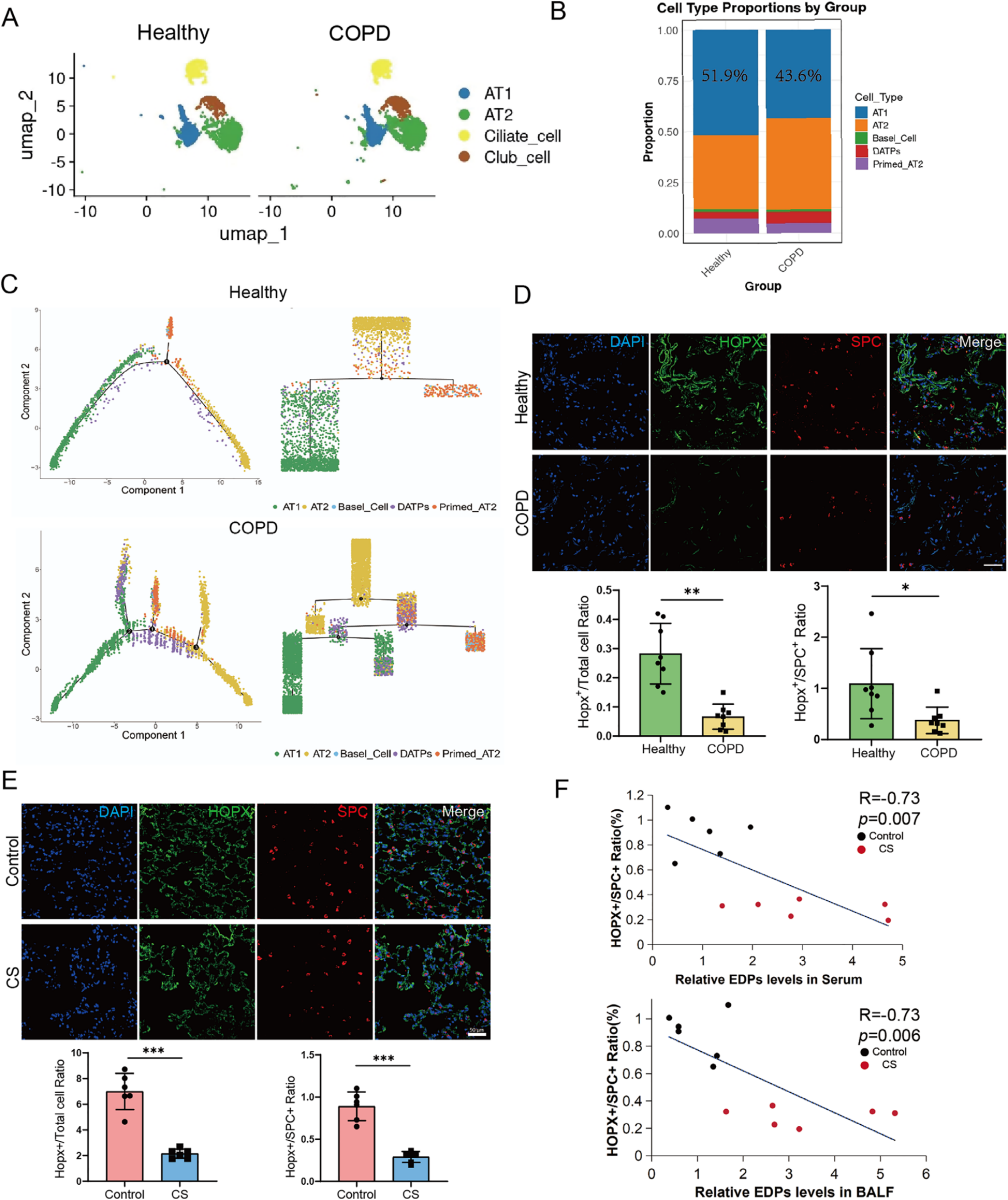
Impaired AT2‐to‐AT1 differentiation in COPD lungs and its association with elevated EDPs levels. (A) Uniform manifold approximation and projection (UMAP) plots showing epithelial cell clusters from healthy donors and patients with COPD. (B) Relative proportions of epithelial cell populations, including alveolar type 1 (AT1) cells, alveolar type 2 (AT2) cells, basal cells, damage‐associated transient progenitors (DATPs), and primed AT2 cells. (C) Pseudotime trajectory analysis of AT2‐to‐AT1 differentiation in healthy and COPD samples. (D) Immunofluorescence staining of human lung sections for HOPX (AT1 marker), surfactant protein C (SPC; AT2 marker), and DAPI, with quantification of the HOPX^+^/total cell ratio and HOPX^+^/SPC^+^ ratio. (E) Immunofluorescence staining of mouse lungs from control and CS‐exposed mice, with quantification of the HOPX^+^/total cell ratio and HOPX^+^/SPC^+^ ratio. (F) Correlation of the HOPX^+^/SPC^+^ ratio with relative EDPs levels in mouse serum (top) and BALF (bottom). Pearson r and *p* values are indicated in the plots. Data are presented as mean ± SEM. Human tissue staining, *n* = 8 per group; mouse staining and correlation analyses, *n* = 6 per group. Panels D and E were analyzed by unpaired two‐tailed Student's *t*‐test. Panel F was analyzed by Pearson correlation. *p < 0.05, **p < 0.01, ***p < 0.001.

To further investigate alveolar epithelial differentiation, we reanalyzed the epithelial‐cell subset from the same human lung scRNA‐seq dataset described in Figure 1A. Uniform manifold approximation and projection (UMAP) analysis identified distinct epithelial cell populations in healthy donors and patients with COPD (Figure 2A) and the marker genes used for cell‐type annotation are shown in Figure S1. Quantitative comparison of cell composition revealed a marked reduction in the proportion of AT1 cells in COPD samples, together with altered proportions of other epithelial subpopulations, including AT2 cells, basal cells, DATPs, and primed AT2 cells (Figure 2B), suggesting alterations in alveolar epithelial cell composition in COPD.

To further define the differentiation status of alveolar epithelial cells, we performed pseudotime trajectory analysis focusing on the AT2‐to‐AT1 transition. In healthy lungs, AT2 cells progressed through intermediate states, including primed AT2 cells and DATPs, before differentiating into mature AT1 cells. In contrast, cells from COPD lungs exhibited an altered differentiation trajectory, characterized by an accumulation of cells in the primed AT2 and DATP states and diminished progression toward the AT1 lineage (Figure 2C), indicating impaired progression along the AT2‐to‐AT1 differentiation trajectory.

We next validated these findings in human lung tissues by immunofluorescence staining for HOPX and surfactant protein C (SPC). Compared with healthy controls, COPD lung tissues exhibited a significant reduction in the HOPX^+^/total cell ratio and the HOPX^+^/SPC^+^ ratio, further supporting impaired AT2‐to‐AT1 differentiation (Figure 2D). Similar alterations were observed in the CS‐exposed mouse model, in which lung tissues from CS‐exposed mice exhibited reduced HOPX expression and a decreased HOPX^+^/SPC^+^ ratio compared with air‐exposed controls (Figure 2E). We next examined whether elevated EDP levels were associated with impaired alveolar epithelial differentiation in vivo. Pearson correlation analysis revealed a strong inverse correlation between the HOPX^+^/SPC^+^ ratio and EDP levels in both serum and BALF from CS‐exposed mice (Figure 2F). These findings suggest that increased EDP abundance is closely associated with defective AT2‐to‐AT1 differentiation.

Collectively, these findings indicate that alveolar epithelial regeneration is compromised in both human COPD and CS‐induced emphysema and reveal a close association between EDP accumulation and impaired AT2‐to‐AT1 differentiation.

### 2.3 CSE and EDPs Impair AT2‐to‐AT1 Differentiation in Mouse and Human Alveolar Organoids

**FIGURE 3:**
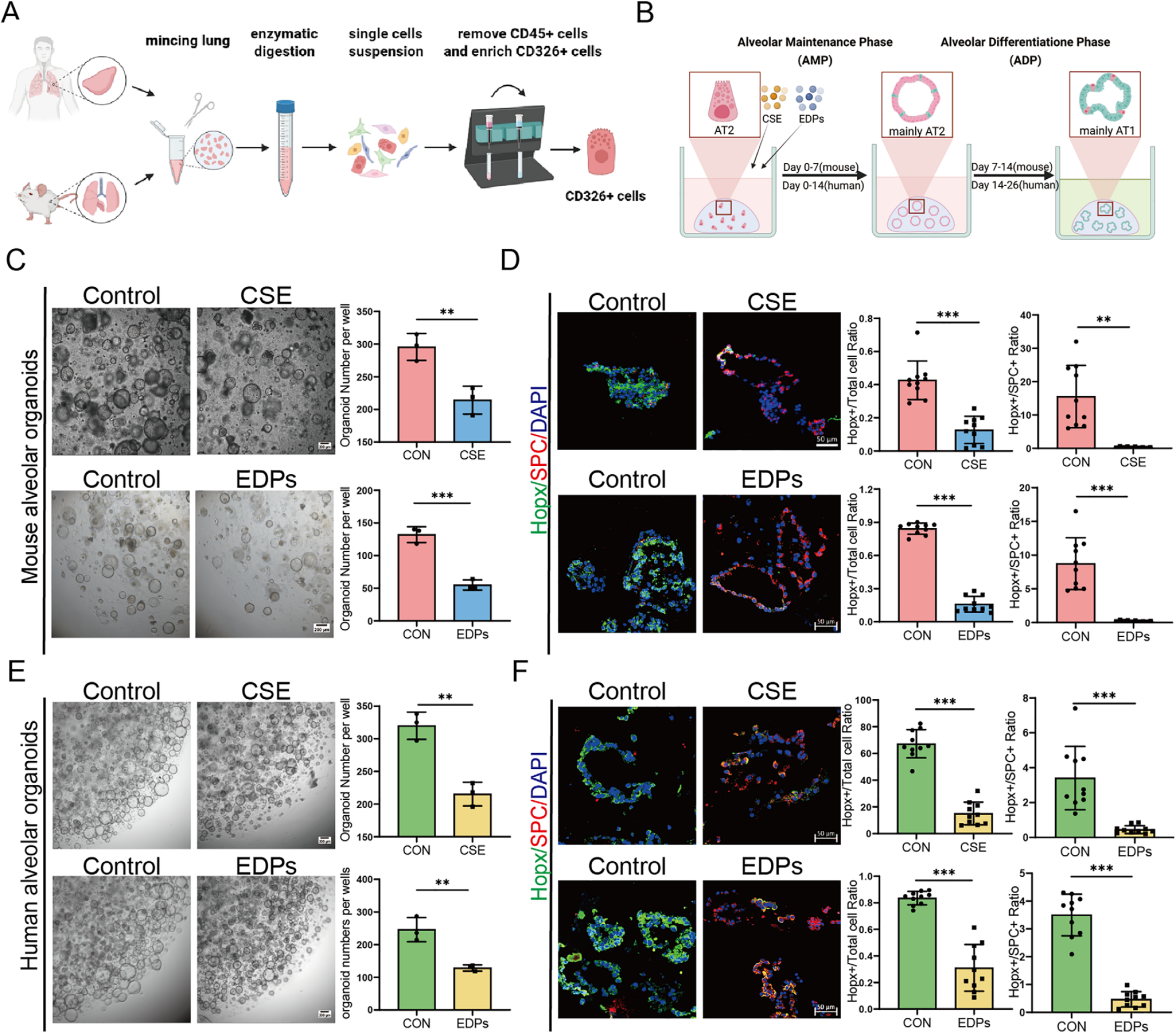
CSE and EDPs impair AT2‐to‐AT1 differentiation in mouse and human alveolar organoids. (A) Schematic workflow for generating alveolar organoids from mouse or human lung epithelial cells enriched for CD326^+^ cells. (B) Schematic of the alveolar maintenance phase (AMP) and alveolar differentiation phase (ADP) in mouse and human organoid cultures. (C) Representative bright‐field images of mouse alveolar organoids treated with cigarette smoke extract (CSE) or elastin‐derived peptides (EDPs), with quantification of organoid number. Scale Bar, 200 µm. (D) Immunofluorescence staining of mouse alveolar organoids for HOPX, SPC, and DAPI, with quantification of the HOPX^+^/total cell ratio and HOPX^+^/SPC^+^ ratio. Scale bar, 50 µm. (E) Representative bright‐field images of human alveolar organoids treated with CSE or EDPs, with quantification of organoid number. Scale bar, 200 µm. (F) Immunofluorescence staining of human alveolar organoids for HOPX, SPC, and DAPI, with quantification of the HOPX^+^/total cell ratio and HOPX^+^/SPC^+^ ratio. Scale bar, 50 µm. Data are presented as mean ± SEM. For organoid‐number assays, *n* = 3 independent experiments. For immunofluorescence quantification, *n* = 10 organoids per group. *p < 0.05, **p < 0.01, ***p < 0.001 by unpaired two‐tailed Student's *t*‐test.

To investigate whether CSE and EDPs directly impair alveolar epithelial regeneration, we established mouse and human alveolar organoid models using protocols adapted from our previously published study [17]. The experimental workflow is summarized in Figure 3A, and organoid development was divided into two phases: the alveolar maintenance phase (AMP), during which AT2 cells proliferate, and the alveolar differentiation phase (ADP), during which AT2 cells undergo differentiation toward the AT1 lineage (Figure 3B).

Based on the dose‐selection experiments shown in Figure S2, mouse organoids were treated with 0.2% CSE or 0.5 mg/mL EDPs, whereas human organoids were treated with 1% CSE or 1 mg/mL EDPs. In mouse alveolar organoids, exposure to either CSE or EDPs significantly reduced organoid number, indicating impaired alveolar organoid growth during the AMP (Figure 3C). At the end of the differentiation phase, immunofluorescence staining for HOPX and SPC showed that both treatments significantly decreased the HOPX^+^/total cell ratio and the HOPX^+^/SPC^+^ ratio, indicating impaired progression toward the AT1 lineage (Figure 3D). We next examined whether similar effects were observed in human alveolar organoids. Consistent with the findings in mouse organoids, both CSE and EDP exposure markedly reduced organoid number in human cultures (Figure 3E). Immunofluorescence analysis further demonstrated significant reductions in both the HOPX^+^/total cell ratio and the HOPX^+^/SPC^+^ ratio following CSE or EDP exposure (Figure 3F), indicating impaired alveolar epithelial differentiation in human organoids.

Collectively, these findings demonstrate that both CSE and EDPs suppress alveolar organoid growth and disrupt AT2‐to‐AT1 differentiation in mouse and human organoid systems, supporting the possibility that EDPs contribute directly to impaired alveolar epithelial regeneration.

### 2.4 EDPs Are Associated With TLR4–NF‐κB Activation and Reduced β‐Catenin Signaling, and TLR4 Inhibition Partially Restores AT2 Differentiation

**FIGURE 4:**
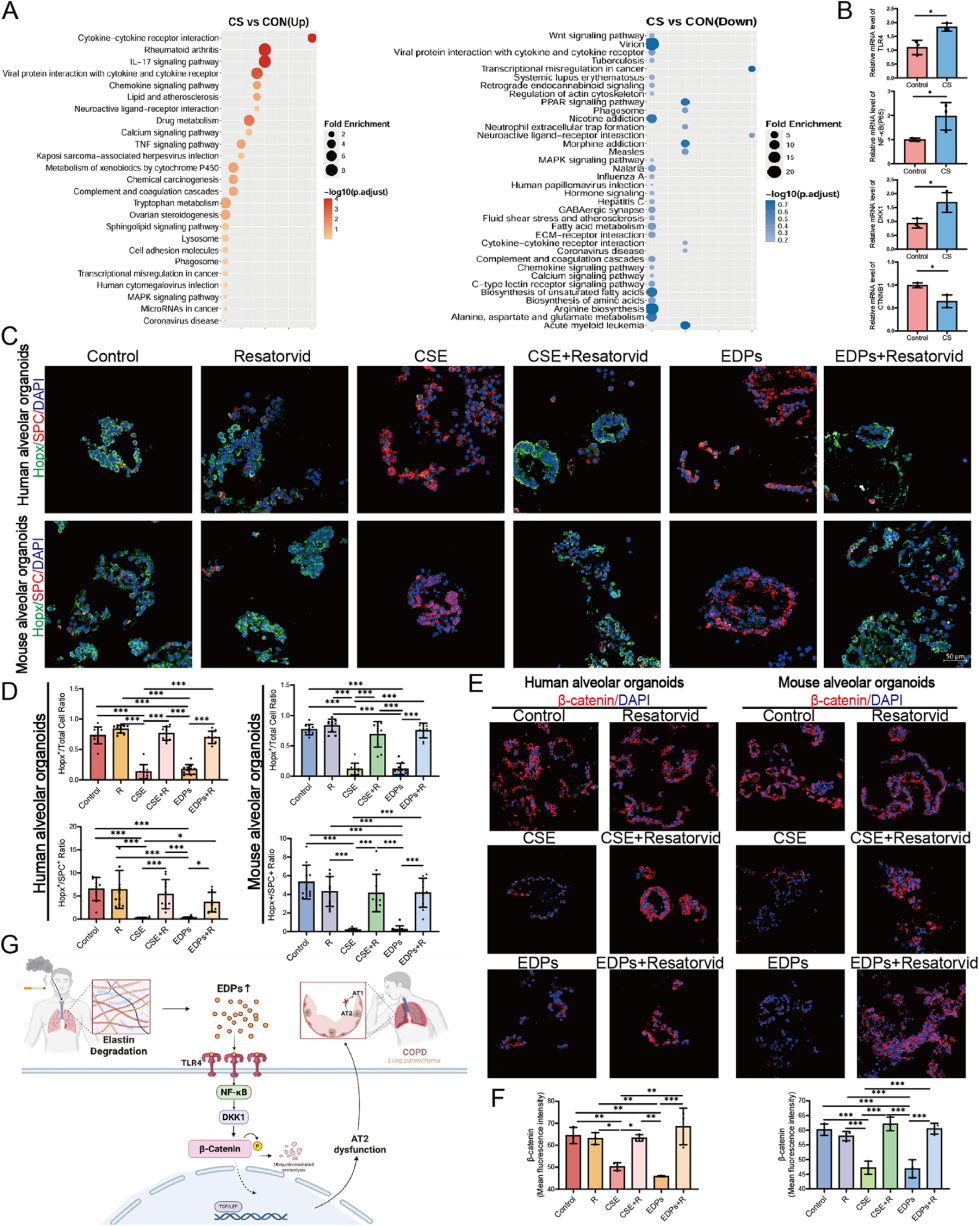
EDPs are associated with TLR4–NF‐κB activation and reduced β‐catenin signaling, and TLR4 inhibition partially restores AT2 differentiation. (A) KEGG enrichment analysis of differentially expressed genes in lungs from control and cigarette smoke (CS)‐exposed mice, showing inflammatory pathways among upregulated terms and Wnt signaling among downregulated terms. (B) Relative mRNA levels of Toll‐like receptor 4 (TLR4), nuclear factor‐κB (NF‐κB; p65), Dickkopf‐1 (DKK1), and CTNNB1 in lung tissue from control and CS‐exposed mice. *n* = 3 mice per group. (C, D) Representative immunofluorescence images of human (top) and mouse (bottom) alveolar organoids stained for HOPX, SPC, and DAPI after treatment with vehicle, resatorvid (R), CSE, CSE + resatorvid, EDPs, or EDPs + resatorvid, with quantification of the HOPX^+^/total cell ratio and HOPX^+^/SPC^+^ ratio. *n* = 10 organoids per group. (E, F) Representative β‐catenin immunofluorescence images and quantification of mean β‐catenin fluorescence intensity in human and mouse alveolar organoids under the indicated treatments. *n* = 10 organoids per group. (G) Proposed working model in which EDPs may activate TLR4/NF‐κB‐related signaling, induce DKK1, reduce β‐catenin signaling, and impair AT2 differentiation; pharmacologic TLR4 inhibition with resatorvid partially alleviates this defect. Data are presented as mean ± SEM. Statistical significance was determined by unpaired two‐tailed Student's *t*‐test in panel B and one‐way ANOVA with Tukey's multiple‐comparison test in panels D and F. *p < 0.05, **p < 0.01, ***p < 0.001.

To explore the molecular mechanisms underlying EDPs‐associated alveolar epithelial dysfunction in COPD, we first performed transcriptomic analysis of lung tissues from mice exposed to CS for 4 months. KEGG pathway enrichment analysis revealed enrichment of inflammatory pathways among upregulated genes, whereas pathways related to stem cell differentiation, including the Wnt signaling pathway, were enriched among downregulated genes in CS‐exposed lungs (Figure 4A). Given prior evidence that EDPs activate NF‐κB‐mediated inflammatory signaling [30, 31] and NF‐κB can suppress β‐catenin pathway activation [32, 33], These concurrent alterations suggested a potential link between enhanced inflammatory signaling and reduced Wnt/β‐catenin‐mediated regenerative activity in COPD‐like lungs. We next examined the expression of key components within these pathways in CS‐exposed lungs.

To further assess this possibility, we performed quantitative PCR analysis of lung tissues from control and CS‐exposed mice. Compared with controls, CS‐exposed lungs showed significantly increased expression of TLR4, NF‐κB (p65), and DKK1, together with reduced expression of CTNNB1 (β‐catenin) (Figure 4B). These transcriptional changes are consistent with enhanced TLR4/NF‐κB‐associated inflammatory signaling and reduced β‐catenin‐related signaling in CS‐induced lung injury.

We next asked whether pharmacological inhibition of TLR4 could alleviate the differentiation defects induced by CSE or EDPs in alveolar organoids. Mouse and human alveolar organoids were treated with control, resatorvid, CSE, CSE + resatorvid, EDPs, or EDPs + resatorvid. Immunofluorescence staining for HOPX and SPC showed that both CSE and EDPs exposure markedly reduced the HOPX^+^/total cell ratio and the HOPX^+^/SPC^+^ ratio, whereas treatment with the TLR4 inhibitor resatorvid partially restored these differentiation‐associated indices in both mouse and human organoids (Figure 4C,D).

We further examined β‐catenin expression under the same treatment conditions. In both mouse and human alveolar organoids, CSE and EDPs exposure reduced β‐catenin expression, whereas resatorvid treatment partially restored β‐catenin expression (Figure 4E,F).

Collectively, these findings support an association between EDP accumulation and activation of TLR4‐associated inflammatory signaling, accompanied by reduced β‐catenin activity. Furthermore, pharmacological inhibition of TLR4 partially rescued both β‐catenin expression and alveolar epithelial differentiation, suggesting a role for TLR4‐associated signaling in mediating the differentiation defects induced by CSE and EDPs (Figure 4G).

### 2.5 Targeted EDP Neutralization by TB Partially Restores Organoid Growth and AT2‐to‐AT1 Differentiation in CSE‐ and EDPs‐Exposed Mouse Alveolar Organoids

We next investigated whether neutralization of EDPs by TB could alleviate the alveolar epithelial defects induced by CSE or EDP exposure in mouse alveolar organoids.

**FIGURE 5:**
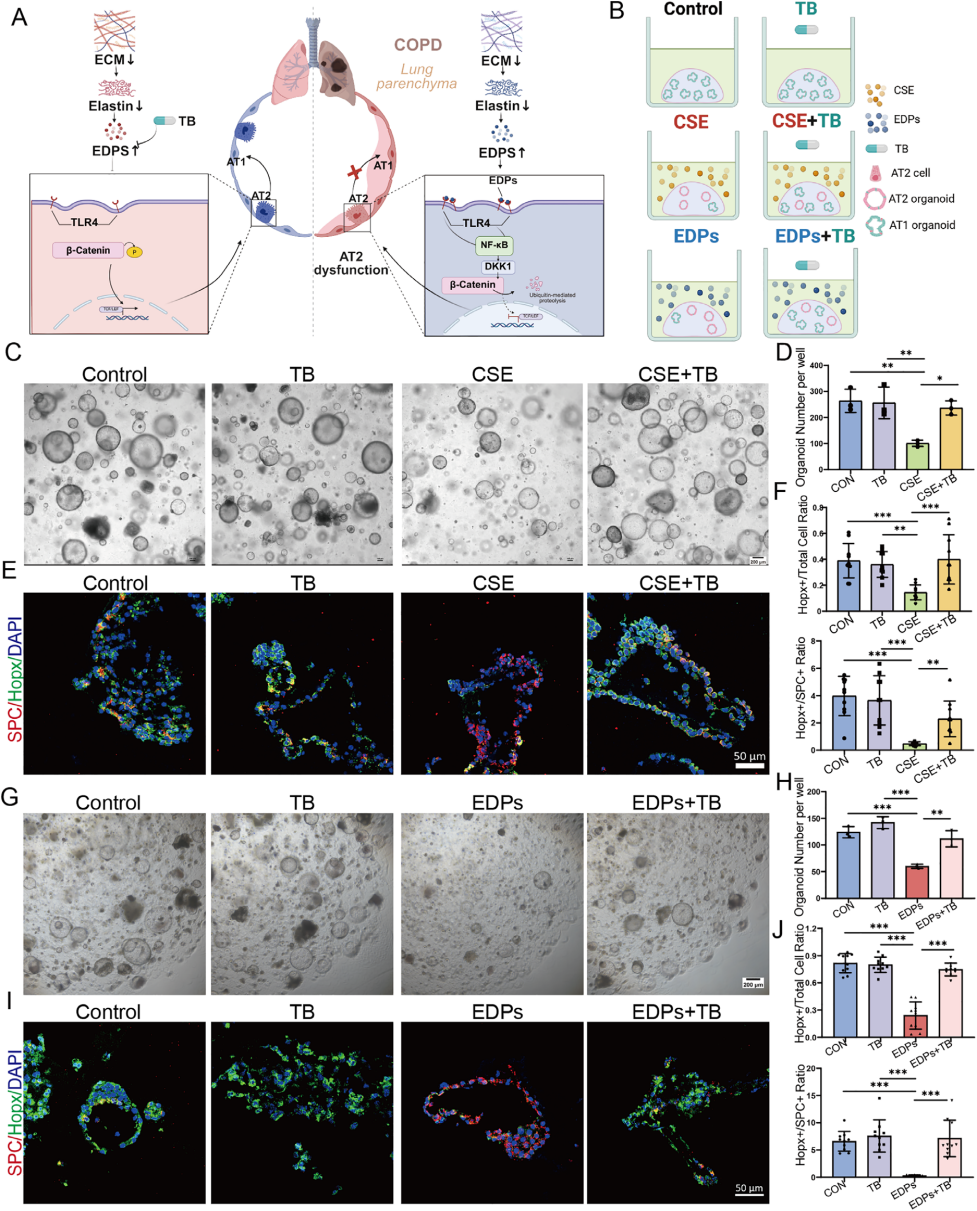
Targeted EDP neutralization by TB partially restores organoid growth and AT2‐to‐AT1 differentiation in CSE‐ and EDPs‐exposed mouse alveolar organoids. (A) Schematic illustration of the proposed mechanism of TB‐B002D (TB) in reversing EDPs‐associated alveolar epithelial dysfunction. (B) Experimental groups included control, TB, CSE, CSE + TB, EDPs, and EDPs + TB. (C, D) Representative bright‐field images of mouse alveolar organoids exposed to CSE with or without TB treatment (C), with quantification of organoid number (D). Scale bar, 100 µm. *n* = 3 independent experiments. (E, F) Immunofluorescence staining of differentiated mouse alveolar organoids (Day 14) showing HOPX (green), SPC (red), and DAPI (blue) in the indicated groups (E), with quantification of the HOPX^+^/total cell ratio and HOPX^+^/SPC^+^ ratio (F). Scale bar, 50 µm. *n* = 10 organoids per group. (G, H) Representative bright‐field images of mouse alveolar organoids exposed to EDPs with or without TB treatment (G), with quantification of organoid number (H). Scale bar, 100 µm. *n* = 3 independent experiments. (I, J) Immunofluorescence staining of differentiated mouse alveolar organoids (Day 14) showing HOPX (green), SPC (red), and DAPI (blue) in the indicated groups (I), with quantification of the HOPX^+^/total cell ratio and HOPX^+^/SPC^+^ ratio (J). Scale bar, 50 µm. *n* = 10 organoids per group. Data are presented as mean ± SEM. *p < 0.05, **p < 0.01, ***p < 0.001 by one‐way ANOVA with Tukey's multiple‐comparison test.

We first examined whether TB could counteract the inhibitory effects of CSE on mouse alveolar organoids. Bright‐field imaging showed that CSE exposure markedly reduced organoid growth, whereas treatment with TB (4 nM) partially restored organoid formation, as evidenced by an increased organoid number (Figure 5C,D). In contrast, TB alone had no obvious effect compared with the control group. Immunofluorescence staining further demonstrated that CSE exposure markedly decreased both the HOPX^+^/total cell ratio and the HOPX^+^/SPC^+^ ratio. Co‐treatment with TB partially reversed these changes, indicating partial restoration of AT2‐to‐AT1 differentiation (Figure 5E,F).

We next investigated whether TB could similarly attenuate the defects caused by direct EDP exposure. Consistent with the findings in the CSE‐treated group, EDP exposure significantly reduced organoid growth, whereas TB treatment partially restored organoid formation capacity (Figure 5G,H). Immunofluorescence analysis showed that EDP treatment also decreased the HOPX^+^/total cell ratio and the HOPX^+^/SPC^+^ ratio, while TB treatment partially restored both indices relative to the EDP‐treated group (Figure 5I,J). TB alone again showed minimal effects compared with untreated controls.

**FIGURE 6:**
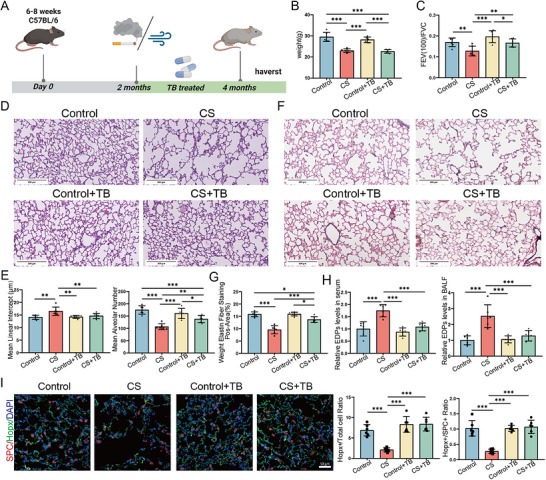
TB treatment ameliorates CS‐induced lung injury and partially restores AT2‐to‐AT1 differentiation in vivo. (A) Schematic overview of the experimental timeline: C57BL/6 mice were exposed to CS for 2 months and then treated with TB for an additional 2 months. Mice were assessed at the end of the 4‐month period for lung function, histopathology, BALF, and serum. (B, C) Body weight (B) and FEV100/FVC ratio (C) measurements in control, CS, CS+TB, and TB‐only groups. TB treatment significantly improved lung function (FEV100/FVC) in CS‐exposed mice. (D) Representative H&E staining of lung tissues from control, CS, and CS+TB groups. TB treatment attenuated the emphysematous changes induced by CS exposure. Scale bar, 200 µm. (E) Quantification of mean linear intercept (MLI) and mean alveolar number in the four experimental groups. TB treatment significantly reduced MLI and restored alveolar density in CS‐exposed mice. (F, G) Elastin fiber staining of lung tissues showing reduced elastic fiber degradation in CS+TB mice compared to CS‐exposed mice. TB treatment preserved lung elastic fiber integrity. Scale bar, 200 µm. (H) Relative EDPs levels in serum and BALF were significantly increased in the CS group compared to control mice, while TB treatment reduced EDPs to levels similar to control. (I) Immunofluorescence staining of lung tissues showing HOPX (green) and SPC (red) expression. TB treatment partially restored AT2‐to‐AT1 differentiation in CS‐exposed mice, as evidenced by the recovery of HOPX^+^/SPC^+^ ratio to control levels. Scale bar, 50 µm. Data are presented as mean ± SEM. *n* = 6 mice per group. *p < 0.05; **p < 0.01; ***p < 0.001 by one‐way ANOVA with Tukey's multiple‐comparison test.

Collectively, these findings demonstrate that TB partially alleviated the impairment of organoid growth and alveolar epithelial differentiation induced by either CSE or EDP exposure. These observations support the concept that excessive EDP accumulation contributes functionally to alveolar regenerative dysfunction and demonstrate that EDP neutralization can improve alveolar epithelial regenerative capacity in vitro. Consistent results were obtained in supplementary analyses showing changes in Ki67^+^/SPC^+^ proliferative AT2 cells (Figure S3).

### 2.6 TB Treatment Ameliorates CS‐Induced Lung Injury and Partially Restores AT2‐to‐AT1 Differentiation in Vivo

To evaluate the therapeutic potential of TB in COPD, TB was administered to mice after 2 months of CS exposure and continued throughout the remaining 2 months of the 4‐month exposure period (Figure 6A). Pulmonary function and lung pathology were assessed at the end of the experiment.

Compared with the CS‐only group, TB administration significantly improved the FEV100/FVC ratio (Figure 6C), indicating partial recovery of CS‐induced pulmonary functional impairment. Histological examination of lung tissues revealed that TB attenuated emphysematous destruction, as evidenced by a reduction in MLI and an increase in alveolar number per field compared with untreated CS‐exposed mice (Figure 6D,E). Further analysis demonstrated that TB partially preserved elastic fiber architecture and increased elastin‐positive area in CS‐exposed lungs (Figure 6F,G). Consistent with these findings, EDP levels in both serum and BALF were markedly elevated following CS exposure but were significantly reduced after TB administration (Figure 6H).

Importantly, no significant differences were observed between the TB‐alone and control groups, indicating that TB treatment did not produce detectable adverse effects under physiological conditions. Immunofluorescence staining further demonstrated that TB increased both the HOPX+/total cell ratio and the HOPX^+^/SPC^+^ ratio in CS‐exposed lungs (Figure 6I), suggesting improved AT2‐to‐AT1 differentiation in vivo. Consistent with the organoid studies, TB treatment also increased the proportion of Ki67^+^/SPC^+^ proliferative AT2 cells in murine lungs (Figure S4). These findings further support a beneficial effect of TB on alveolar epithelial regeneration following chronic CS exposure. Notably, while all‐trans retinoic acid (ATRA) has been proposed as a candidate therapy for emphysema [34], TB produced greater overall improvement under the present experimental conditions, particularly with respect to pulmonary function (Figure S5).

Collectively, these findings demonstrate that TB alleviates CS‐induced emphysematous lung injury, reduces EDP accumulation, and partially restores alveolar epithelial regenerative capacity in vivo, supporting the therapeutic potential of EDP neutralization as a strategy for COPD treatment.

## 3 Discussion

This study identifies EDPs as a previously underrecognized matrix‐derived factor associated with impaired alveolar regeneration in COPD. By integrating analyses of human lung tissues, CS‐exposed mouse models, and mouse and human alveolar organoids, we found that elevated EDP levels were associated with elastic fiber disruption and impaired AT2‐to‐AT1 differentiation. In addition, pharmacological neutralization of EDPs with TB partially restored alveolar epithelial differentiation in organoids, ameliorated emphysematous changes, and improved pulmonary function in CS‐exposed mice. Collectively, these findings support the concept that elastin degradation contributes to defective alveolar repair in COPD and suggest that EDP‐targeted intervention warrants further investigation as a potential regenerative therapeutic strategy.

The ECM plays a fundamental role in maintaining pulmonary elasticity and normal respiratory function [35]. Although ECM remodeling has long been recognized as a hallmark of COPD [36, 37, 38], most previous studies addressing impaired epithelial regeneration have primarily provided descriptive observations [16, 17], without clearly identifying the upstream ECM‐derived mediator linking tissue destruction to progenitor‐cell dysfunction. In this context, our study extends prior work on defective AT2‐to‐AT1 transition in COPD [16, 17] by identifying EDPs as a potential pathogenic mediator that may connect elastin degradation to impaired alveolar epithelial differentiation [24, 25]. Importantly, this perspective extends beyond descriptive observations of regenerative failure and offers a biologically plausible link between ECM destruction and epithelial dysfunction.

Our findings further suggest that TLR4‐associated inflammatory signaling may contribute to EDP‐associated alveolar epithelial dysfunction. In the present study, KEGG enrichment analysis indicated enhanced inflammatory signaling together with reduced Wnt‐related regenerative activity, and we observed increased expression of TLR4, NF‐κB, and DKK1, accompanied by reduced β‐catenin expression, in CS‐exposed lungs. Moreover, the TLR4 inhibitor resatorvid partially rescued the differentiation defects induced by CSE or EDPs and partially restored β‐catenin expression, providing pharmacological evidence supporting a role for TLR4‐associated signaling. However, these data do not conclusively establish causality for the entire TLR4/NF‐κB/Wnt/β‐catenin axis. Our focus on TLR4 was guided by prior literature linking EDPs to TLR4‐related inflammatory signaling [30, 31, 32], together with the TLR4‐associated changes observed in our models. Nevertheless, additional inflammatory pathways, including TNF‐related signaling, may also contribute and warrant further investigation [39, 40]. In addition, previous studies have suggested that limiting excessive inflammatory signaling may favor Wnt‐associated regenerative responses in the lung [41], which is broadly consistent with our observations. Because sustained inflammation, immune‐cell infiltration, and progressive ECM degradation are closely linked during COPD progression [18, 22, 42, 43], our findings raise the possibility that EDPs accumulation contributes to a potential self‐amplifying cycle involving inflammation, matrix remodeling, and impaired alveolar regeneration.

Given the potential limitations of broad suppression of upstream inflammatory pathways, including possible suppression of innate immunity and associated safety concerns during long‐term treatment [44], TB represents a potentially attractive translational approach because it is designed to neutralize EDPs and may offer a more selective strategy for interrupting pathological EDP signaling [29]. In our study, TB partially restored organoid growth and AT2‐to‐AT1 differentiation in CSE‐ and EDPs‐exposed organoids and also improved emphysematous pathology in vivo. These findings suggest that targeting EDPs may help disrupt pathological processes that sustain inflammation and compromise alveolar regeneration. Nevertheless, the molecular specificity of TB in the context of COPD has not yet been fully defined, and its long‐term safety, durability of efficacy, optimal route of administration, and translational feasibility remain to be established.

Several limitations merit consideration. First, the clinical sample size was modest, including only eight patients with COPD in the current analysis. Second, although the organoid and inhibitor experiments strengthen the biological relevance of our findings, the downstream signaling mechanism was only partially addressed, and additional genetic and mechanistic studies will be required to establish causal signaling relationships. Third, the therapeutic effects of TB were evaluated primarily in a short‐term preclinical setting. Fourth, the human serum and BALF samples used for EDP measurement were obtained from independent, non‐paired clinical cohorts and were not matched to the surgical tissue cohort used for histological analysis; therefore, the human data should be interpreted primarily as group‐level associations rather than individual‐level correlations.

Overall, our study identifies EDPs as previously underrecognized mediators linking ECM degradation to impaired alveolar regeneration in COPD. By demonstrating that pharmacological neutralization of EDPs partially restores alveolar epithelial differentiation and attenuates emphysematous injury, our findings support further investigation of EDP‐targeted approaches as a potential regenerative strategy for promoting alveolar repair in chronic lung disease.

## 4 Materials and Methods

### 4.1 Patients

Eight patients with COPD and eight healthy controls without airflow obstruction were recruited for this study (Table S1). The study was approved by the Ethics Committee of Guangzhou Medical University (Approval No. 202503026).

### 4.2 Animals and Treatments

Male C57BL/6J mice (6–8 weeks old) were purchased from Shanghai Model Organisms and maintained under specific pathogen‐free (SPF) conditions. All animal procedures were approved by the Institutional Animal Care and Use Committee of Guangzhou Medical University (No. 2022–089).

TB‐B002D (TB), a peptide drug provided by Shenzhen Turier Biotech Co., Ltd., was dissolved in phosphate‐buffered saline (PBS) before use. Mice were randomly assigned to four groups (*n* = 6 per group): [1] air control [2], CS exposure [3], TB treatment alone (80 µg/kg/day) [4], and CS exposure plus TB treatment.

CS exposure was conducted using a whole‐body exposure system with filter cigarettes. Following a 7‐day progressive adaptation period (6–12 cigarettes per session), mice were exposed to CS for 4 months (four sessions per day, 12 cigarettes per session). TB was administered via intraperitoneal injection (i.p.) during months 3–4. Pulmonary function testing and tissue collection were performed at the experimental endpoint.

### 4.3 Lung Function Measurement

Pulmonary function was assessed using a Pulmonary Function Test system (DSI Buxco) under anesthesia induced by intraperitoneal tribromoethanol (2.5% avertin, 10–20 µL/g). Mice were intubated and mechanically ventilated for the measurement of functional residual capacity (FRC), forced vital capacity (FVC), quasi‐static pressure–volume (PV) curves, airway resistance, and compliance.

### 4.4 Collection of Serum and BALF

Peripheral blood was collected at sacrifice and centrifuged at 3000 × *g* for 15 min to obtain serum. For mouse BALF collection, the trachea was cannulated and the lungs were lavaged five times with sterile PBS, with a total lavage volume of 3 mL. The recovered BALF was centrifuged to remove cellular debris, and the supernatant was collected for subsequent EDPs measurement.

Human BALF was collected by bronchoscopy. Human BALF and serum samples used for EDPs measurement were obtained from independent, non‐paired clinical cohorts and were not matched to the surgical tissue cohort used for lung tissue analysis. BALF samples were centrifuged to remove cellular debris, and the supernatants were used for subsequent EDPs measurement.

### 4.5 Measurement of EDPs

EDPs levels in serum and BALF were measured using a commercial EDP detection kit (Biocolor, F2000) according to the manufacturer's instructions. Absorbance was measured at 513 nm using a microplate reader (Tecan). Because serum contains abundant interfering substances that may increase background signals, EDPs levels were normalized to the corresponding control group and are presented as relative levels rather than absolute concentrations.

### 4.6 Preparation of cigarette smoke extract (CSE)

CSE was prepared by combusting commercial cigarettes using a programmable smoking apparatus integrated with a small‐animal ventilator. Smoke was passed through three sequential PBS‐filled condensers (5 mL each) under controlled conditions. The resulting suspension was sterilized through a 0.22‐µm filter (Biosharp, BS‐PES‐22), aliquoted as 100% CSE stock, and stored at −80°C until use.

### 4.7 Lung Cell Isolation and Epithelial‐Cell Enrichment

Primary mouse lung epithelial cells were isolated from adult C57BL/6 mice by enzymatic digestion and magnetic‐activated cell sorting (MACS). Briefly, after systemic PBS perfusion, lung tissues were minced and digested in DMEM/F12 containing collagenaseI(2 mg/mL) and DNase (0.05 mg/mL) at 37°C with gentle agitation. Digested suspensions were filtered through a 40‐µm cell strainer, treated with red blood cell (RBC) lysis buffer when necessary, and subjected to CD45‐positive cell depletion followed by CD326‐positive epithelial‐cell enrichment for subsequent organoid culture.

For human samples, lung tissues were processed as soon as possible after collection. After removal of visible blood vessels and connective tissue, the tissues were minced into small fragments and digested in DMEM/F12 containing DNase (0.05 mg/mL), dispase (0.8 mg/mL), and collagenaseI (2 mg/mL) at 37°C with gentle agitation. The resulting cell suspension was filtered through a 40‐µm cell strainer, treated with RBC lysis buffer when necessary, and then subjected to CD45‐positive cell depletion followed by CD326‐positive epithelial‐cell enrichment before organoid culture.

Thus, the human and mouse isolation workflows were similar in principle but not identical. The major differences were the enzyme composition used for tissue digestion, the handling of clinical tissue samples, and the subsequent culture conditions used for human and mouse organoids.

### 4.8 Organoid Culture

Mouse and human alveolar organoids were generated using similar overall procedures, but their culture conditions were not identical. Freshly sorted epithelial cells were resuspended in alveolar maintenance medium and mixed with growth factor‐reduced Matrigel (Corning, 356234). A total of 5 × 10^3^ CD45^−^CD326^+^ epithelial cells and 5 × 10^4^ CD45^−^CD326^−^ supporting cells were plated as 50 µL droplets in 12‐well plates.

For mouse organoids, the AMP was performed from Day 0 to 7, followed by the ADP from Day 7 to 14. For human organoids, the AMP was performed from Day 0 to 14, followed by the ADP from Day 14 to 28. Detailed formulations of the mouse and human alveolar maintenance and differentiation media are provided in Tables S2 and S3.

### 4.9 Organoid Treatment With CSE, EDPs, TB, and Resatorvid

For CSE treatment, CSE was added at 0.2% for murine organoids and 1% for human organoids. EDPs were dissolved in PBS and added at 0.5 mg/mL for murine organoids and 1 mg/mL for human organoids. Control organoids received the corresponding medium without CSE or EDPs. For rescue experiments, organoids were treated with TB (4 nM) or the TLR4 inhibitor resatorvid (1 nM). TB or resatorvid was added using the same treatment schedule as CSE or EDPs exposure.

### 4.10 RNA Extraction and Quantitative PCR

Total RNA was extracted using Nucleozol reagent (MNG, 740404) according to the manufacturer's instructions. Complementary DNA was synthesized using Evo M‐MLV reverse transcriptase. Quantitative PCR was performed using SYBR Green Pro Taq HS mix (Accurate Bio., AG11707) on a CFX96 thermocycler (Bio‐Rad). Gene expression was normalized to β‐actin and calculated using the 2^−ΔΔCt^ method. Primer sequences are provided in Table S4.

### 4.11 Elastic Fiber Staining

Lung tissues were fixed in 4% paraformaldehyde, dehydrated through graded ethanol, embedded in paraffin, and sectioned. Sections were stained using an Elastic Van Gieson (EVG) kit (Baso Diagnostics). Whole‐slide imaging was performed using a Leica Aperio CS2 scanner (20×/0.75 NA, 0.45 µm/pixel). Quantitative morphometric analysis was conducted in ImageJ using Otsu thresholding.

### 4.12 Histology and Immunofluorescence

For histological analysis, lung tissues were fixed overnight at 4°C in 4% paraformaldehyde, embedded in paraffin, sectioned, and stained with H&E.

For immunofluorescence, fresh lung tissues were fixed for 24 h in 4% paraformaldehyde, dehydrated sequentially in 10%, 20%, and 30% sucrose at 4°C, and embedded in optimal cutting temperature (OCT) compound. Cryosections (8 µm) were equilibrated at room temperature for 10 min, permeabilized with 0.3% Triton X‐100 for 15 min, washed three times with PBS, and blocked with 10% bovine serum containing 0.1% Tween‐20 for 1 h at room temperature. Sections were then incubated overnight at 4°C with primary antibodies including anti‐pro‐SPC (rabbit polyclonal, 1:1000, Millipore AB3786), anti‐HOPX (mouse polyclonal, 1:100, Santa Cruz sc‐398703), anti‐β‐catenin (rabbit polyclonal, 1:250, Proteintech 51067‐2‐AP), and anti‐Ki67 (rat polyclonal, 1:100, Invitrogen 14‐5698‐95). After washing, sections were incubated with species‐matched Alexa Fluor‐conjugated secondary antibodies (1:1000, Invitrogen) for 1 h at room temperature. Nuclei were counterstained with DAPI (1:1000, Biofroxx) for 10 min. Images were acquired using a Zeiss LSM 800 confocal microscope.

### 4.13 Single‐Cell RNA‐seq Data Processing and Pseudotime Analysis

Single‐cell RNA‐seq data were processed and analyzed using Seurat (v5.0.2). Cells were retained after quality control if they met the following criteria: mitochondrial gene proportion < 10% (percent.mt < 10%) and detected gene number between 200 and 5000 (200 < nFeature_RNA < 5000). Gene expression data were normalized using the LogNormalize method with a scale factor of 10,000. A total of 3000 highly variable genes were identified using the vst method and used for downstream analyses. Principal component analysis (PCA) was performed on the normalized data, and Harmony (v1.2.4) was applied to integrate data across different batches and/or species to reduce batch effects. UMAP was performed for visualization based on the first 15 Harmony‐corrected principal components. Cell clustering was conducted using the first 20 principal components with a resolution parameter of 0.2. Cell clusters were annotated according to canonical marker genes.

Pseudotime trajectory analysis of epithelial cells was performed using Monocle2. Highly variable genes were selected as ordering genes, and dimensionality reduction and trajectory construction were carried out using the DDRTree algorithm. AT2 cells were defined as the root state for trajectory inference. Genes that changed significantly along pseudotime were identified using the differentialGeneTest function with a cutoff of *q* < 0.01.

### 4.14 Statistical Analysis

Statistical analyses were performed using GraphPad Prism 9.0. Unpaired two‐tailed Student's *t*‐test was used for comparisons between two groups, whereas one‐way ANOVA followed by Tukey's multiple‐comparison test was used for comparisons among multiple groups, as specified in the figure legends. The number of biological replicates is indicated in the corresponding legends. Data are presented as mean ± SEM. A value of *p* < 0.05 was considered statistically significant; significance levels are indicated as *p < 0.05, **p < 0.01, and ***p < 0.001.

## Author Contributions

**Jianwei Dai**: supervision, project administration, funding acquisition. **Pixin Ran**: conceptualization, resources, supervision. **Bingjie Chen**: writing – original draft, project administration. **Fei Cui**: resources, investigation. **Huijuan Zhu**: writing – original draft, validation, methodology. **Yiling Zhao**: conceptualization. **Yingchao Qin**: methodology, formal analysis. **Wenfeng Huang**: validation, methodology. **Jiahong Kuang**: methodology, formal analysis. **Zihan Liu**: formal analysis, data curation. **Jiarui Weng**: formal analysis, data curation. **Zibei Feng**: validation, methodology. **Zhilian Ye**: validation, methodology. **Peiji Zheng**: formal analysis, data curation. **Xiaolan Guo**: project administration, validation. All authors reviewed and approved the final manuscript.

## Funding

This work was supported by the National Natural Science Foundation of China (32270790), Major Project of Guangzhou National Laboratory (GZNL2023A02006), Guangdong Provincial Natural Science Foundation (2025A1515011337), Project of State Key Laboratory of Respiratory Disease (SKLRD‐Z‐202402), and Guangzhou National Laboratory and State Key Laboratory of Respiratory Disease (GZNL2025B01010).

## Conflicts of Interest

The authors declare no conflicts of interest.

## Ethics Statement

Animal procedures were approved by the Institutional Animal Care and Use Committee of Guangzhou Medical University (Approval No. 2022–089). Clinical studies were approved by the Ethics Committee of Guangzhou Medical University (Approval No. 202503026).

## Supporting information




**Supporting File 1**: mco270889‐supp‐0001‐SuppMat.docx

## Data Availability

The sequencing data have been deposited in the National Genomics Data Center, China National Center for Bioinformation (CNCB‐NGDC), under BioProject ID PRJCA039937. Raw sequencing data are available in the OMIX database under accession number OMIX011804.
