# Supplementary material for: Targeting Elastin‐Derived Peptides Reverses Alveolar Epithelial Dysfunction in Chronic Obstructive Pulmonary Disease
Source: MedComm (2020). 2026 Jul 25;7(8):e70889. doi: 10.1002/mco2.70889 (PMC13401145; doi:10.1002/mco2.70889)
Supplement: Supplementary file 1 — Supporting File 1: mco270889‐supp‐0001‐SuppMat.docx [file MCO2-7-e70889-s001.docx]

Targeting elastin-derived peptides reverses alveolar epithelial dysfunction in chronic obstructive pulmonary disease (COPD)

Huijuan Zhu^1,2,3,^#, Yiling Zhao^1,2,^#, Yingchao Qin^1,2,^#, Wenfeng Huang^1,2^, Jiarui Weng^1,2^, Zihan Liu^1,2^, Jiahong Kuang^1,2^, Zibei Feng^1,2^, Zhilian Ye^1,2^, Peiji Zheng^1,2^, Xiaolan Guo^1,2^, Fei Cui^3^*, Bingjie Chen^1,2,3^*, Pixin Ran^3^*, Jianwei Dai^1,2,3^*

1 GMU-GIBH Joint School of Life Sciences, The Guangdong-Hong Kong-Macao Joint Laboratory for Cell Fate Regulation and Diseases, State Key Laboratory of Respiratory Disease, Guangzhou Medical University

2 Department of Intensive Care Unit, the Second Affiliated Hospital, Guangzhou Medical University, Guangzhou, 510260, Guangdong, P.R.China

3 Department of Intensive Care Unit, the First Affiliated Hospital, Guangzhou Medical University, Guangzhou, 510120, Guangdong, P.R.China

4 Guangzhou National Laboratory, Bio-Island, Guangzhou, P.R.China

# These authors contributed equally to this work.

*Corresponding author: Jianwei Dai, Bingjie Chen, Fei Cui

E-mails: [daijw@gzhmu.edu.cn](mailto:daijw@gzhmu.edu.cn); [pxran@gzhmu.edu.cn;](mailto:pxran@gzhmu.edu.cn;) [bingjiechen@gzhmu.edu.cn](mailto:bingjiechen@gzhmu.edu.cn); [cuidavil@hotmail.com](mailto:cuidavil@hotmail.com)

**Supplementary Information**

**Table S1:**

Demographic Data.

Demographics and pulmonary function test results from Healthy and COPD subjects. (n) = number of subjects with available data. For continuous variables, data reflects mean ± standard deviation).

|  | **Healthy** | **COPD** |
| --- | --- | --- |
| **Number of subjects** | 8 | 8 |
| **Sex** |  | |
| female | 2 | 1 |
| male | 6 | 7 |
| Age | 68.75±8.2 | 68.88±6.7 |
| **Ever smoker** | 3 | 3 |
| **Lung function** |  | |
| FEV1(%) | - | 61.62±6.7 |
| FEV1/FVC | 80.2±3.9 | 49.8±4.4 |
| MMEF75/25(%) | - | 15.3±3.8 |
| MVV(%) | - | 66.4±9.8 |

**Table S2:**

**Alveolar Maintenance Medium (AMM) for murine culture**

| Reagent | Final concentration |  |
| --- | --- | --- |
| SB-431542 | 10μM | MCE HY-10431 |
| CHIR-99021 | 3μM | MCE HY-10182G |
| Y-27632 | 10μM | MCE HY-10071 |
| NOGGIN | 10 ng/mL | MCE HY-P7051A |
| BIRB796 | 1μM | MCE HY-10320 |
| DMH-1 | 1μM | MCE [HY-12273](https://www.medchemexpress.cn/DMH-1.html) |
| mFGF-7 | 10 ng/mL | MCE HY-P7176 |
| mFGF-10 | 10 ng/mL | MCE HY-P7170 |
| mEGF | 50 ng/mL | MCE [HY-P7067](https://www.medchemexpress.cn/recombinant-proteins/egf-protein-mouse.html) |
| RSpondin-1 | 10ng/mL | MCE HY-P76012 |
| B27 supplement | 1X | Gibco 17504044 |
| Antibiotic antimycotic (Anti-Anti) | 1X | ECOTOP ES-8440 |
| Advanced DMEM/F12 | - | Gibco 12634010 |

**Alveolar Differentiation Medium (ADM) for murine culture**

| Reagent | Final concentration |  |
| --- | --- | --- |
| FBS | 10% | Excell FSP500 |
| mFGF-10 | 1 ng/mL | MCE HY-P7170 |
| mEGF | 5 ng/mL | MCE [HY-P7067](https://www.medchemexpress.cn/recombinant-proteins/egf-protein-mouse.html) |
| XAV-939 | 10μM | MCE [HY-15147](https://www.medchemexpress.cn/XAV-939.html) |
| Antibiotic antimycotic (Anti-Anti) | 1X | ECOTOP ES-8440 |
| Advanced DMEM/F12 | - | Gibco 12634010 |

**Table S3:**

**Alveolar Maintenance Medium (AMM) for human culture**

| Reagent | Final concentration |  |
| --- | --- | --- |
| SB-431542 | 10μM | MCE HY-10431 |
| CHIR-99021 | 3μM | MCE HY-10182G |
| Y-27632 | 10μM | MCE HY-10071 |
| BIRB796 | 1μM | MCE HY-10320 |
| hFGF-7 | 10 ng/mL | MCE HY-P70597 |
| hFGF-10 | 10 ng/mL | MCE HY-P78123 |
| hEGF | 50 ng/mL | MCE HY-P7109 |
| B27 supplement | 1X | Gibco 17504044 |
| Antibiotic antimycotic (Anti-Anti) | 1X | ECOTOP ES-8440 |
| Advanced DMEM/F12 | - | Gibco 12634010 |

**Alveolar Differentiation Medium (ADM) for human culture**

| Reagent | Final concentration |  |
| --- | --- | --- |
| Human serum | 10% | Sigma-Aldrich H4522 |
| hFGF-10 | 1 ng/mL | MCE HY-P78123 |
| hEGF | 5 ng/mL | MCE HY-P7109 |
| XAV-939 | 10μM | MCE [HY-15147](https://www.medchemexpress.cn/XAV-939.html) |
| Antibiotic antimycotic (Anti-Anti) | 1X | ECOTOP ES-8440 |
| Advanced DMEM/F12 | - | Gibco 12634010 |

**Table S4: Primer sequences used in this study.**

| **Primer** | **Sense (5’to3’)** | **Anti-Sense (5’to 3’)** |
| --- | --- | --- |
| Mouse-TLR4 | ACCTCAGCTTCAATGGTGCC | GCTGAAAATCCAGGTGCTGC |
| Mouse- NF-κB（P65） | GGCCTCATCCACATGAACTT | CACTGTCACCTGGAAGCAGA |
| Mouse-DKK1 | CCCTCTGACCACAGCCATTT | GGTGCACACCTGACCTTCTT |
| Mouse-CTNNB1 | ACTTGCCACACGTGCAATTC | AAGGTTGTGCAGAGTCCCAG |
| Mouse- β-actin | TATAAAACCCGGCGGCGCA | GTCATCCATGGCGAACTGGTG |

**Figure S1:**


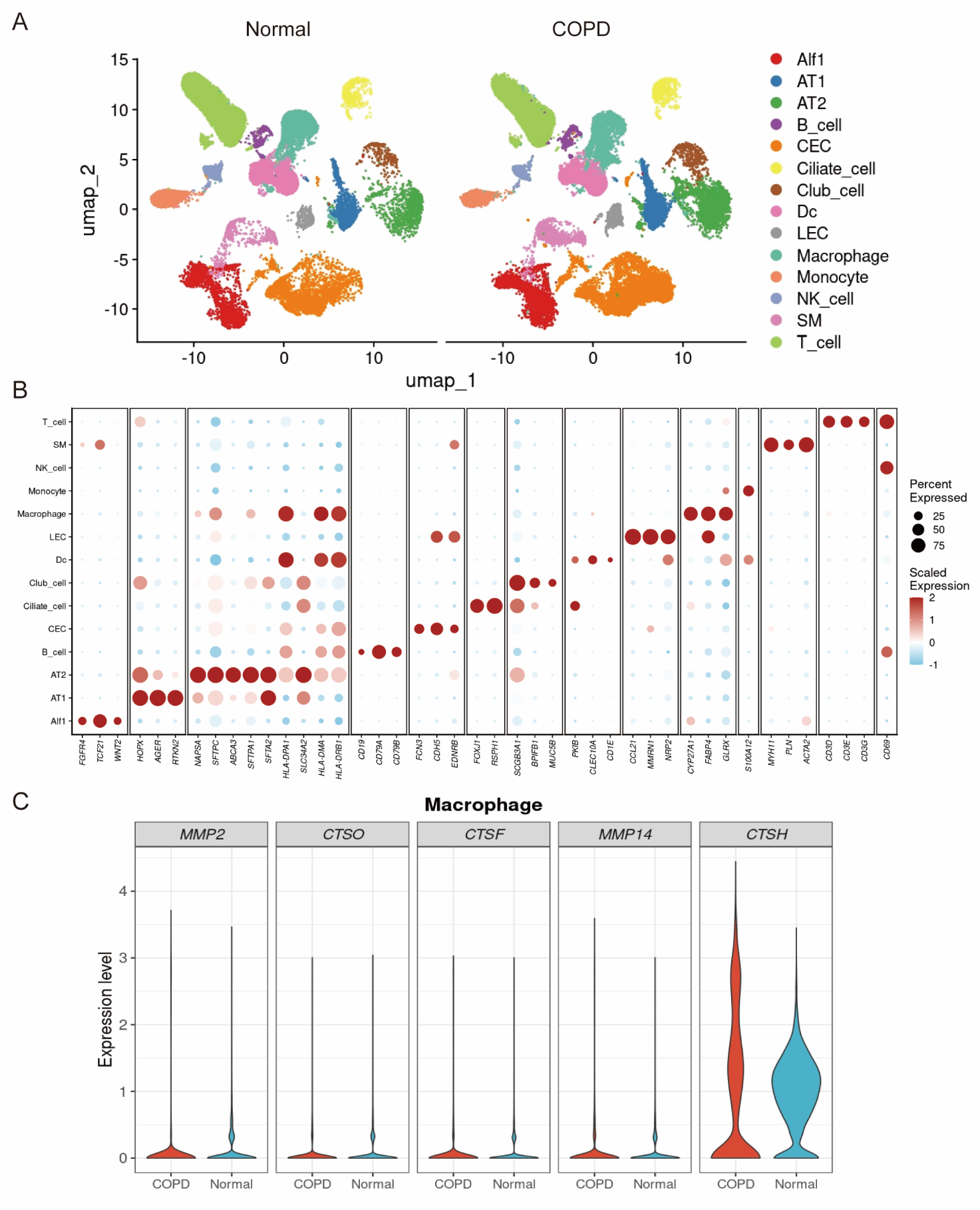


**Fig. S1. (A)** UMAP plots. Display cell type clustering in healthy (left) and COPD (right). Different colors denote cell types. **(B)** Dotplot heatmap showing select genes used to annotate cell types in the dataset. Marker genes used for epithelial cell annotation were as follows: AT1 cells (HOPX, AGER, RTKN2), AT2 cells (SFTPC, SFTPA1, SFTA2, ABCA3), ciliated cells (FOXJ1, RSPH1), and club cells (BPIFB1, SCGB3A1, MUC5B). **(C)** Transcriptomic analysis revealed significant upregulation of ECM-degrading enzymes, in case of MMP2, CTSO, CTSF, MMP14, CTSH.

Figure S2:


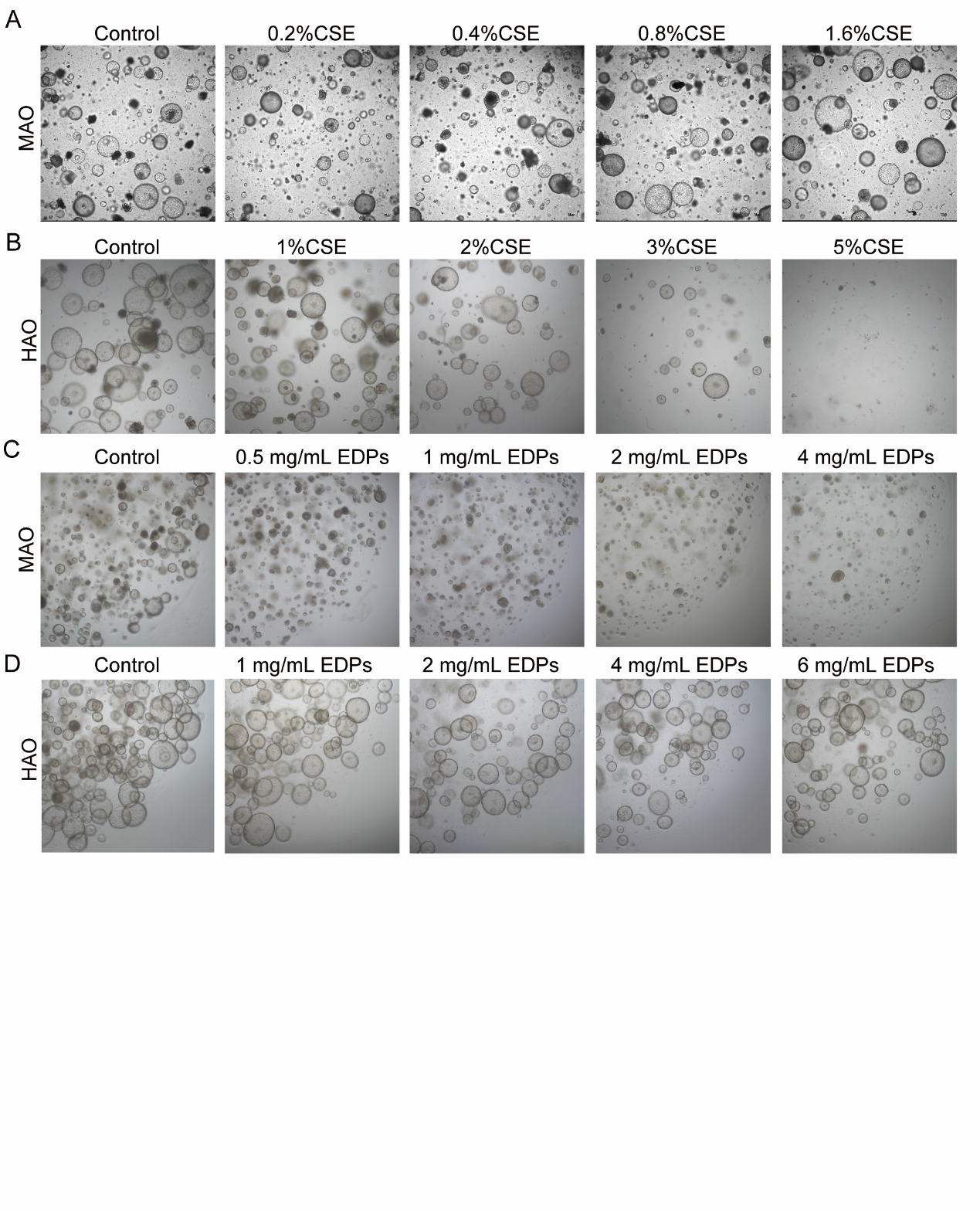


**Fig. S2.** **(A)** Brightfield images of mouse alveolar organoids (MAO) exposed to CSE. **(B)** Brightfield images of human alveolar organoids (HAO) exposed to CSE. **(C)** Brightfield images of mouse alveolar organoids exposed to EDPs. **(D)** Brightfield images of human alveolar organoids exposed to EDPs.

Figure S3:


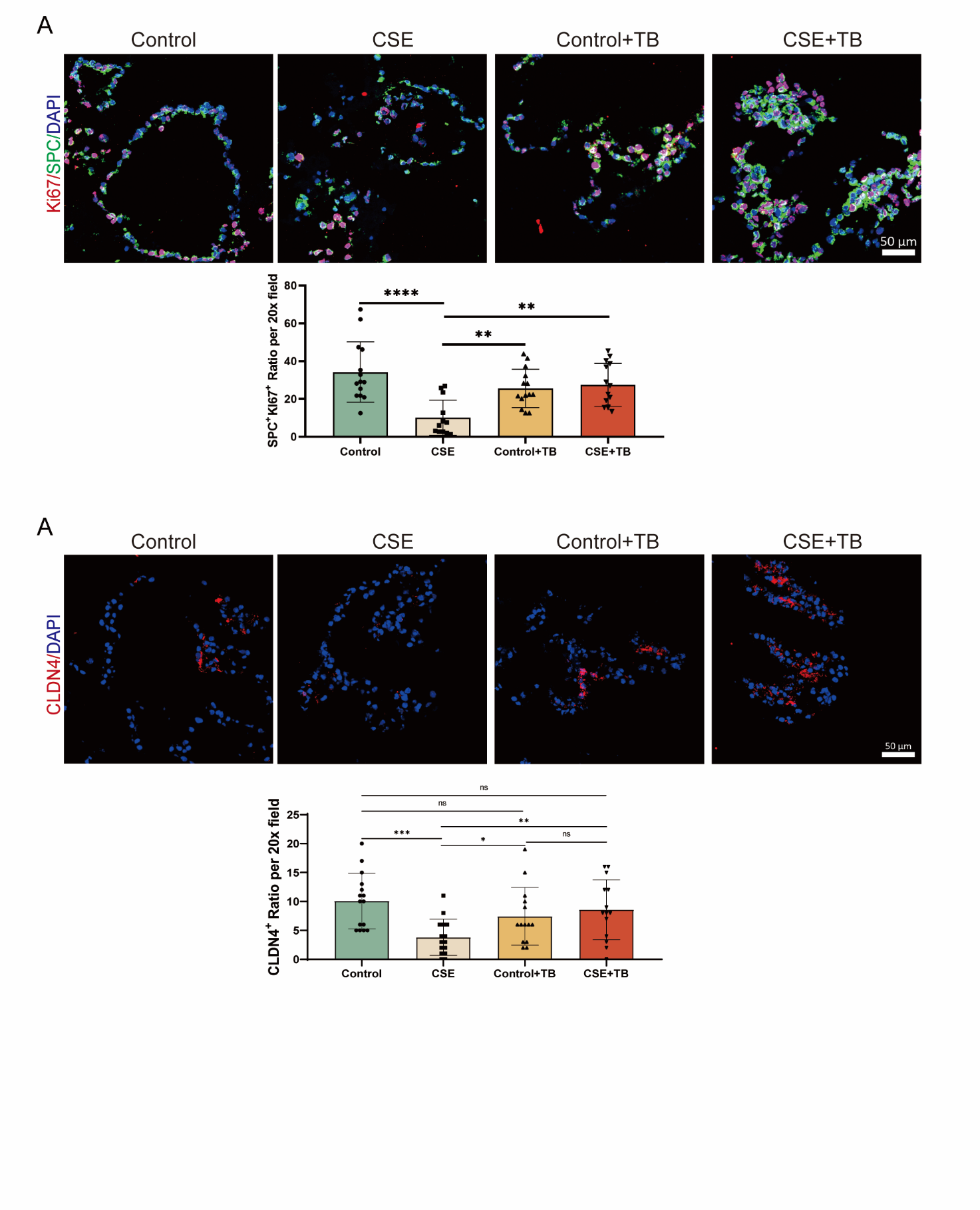


**Fig. S3.** **(A)** Immunofluorescence staining of the Alveolar Maintenance Phase (Day 7) showing SPC (AT2 marker, green), Ki67 (red), and DAPI (blue) in Control, CSE, Control+TB, CSE+TB groups. And quantification of SPC^+^ /Ki67^+^ ratios indicates that TB treatment promotes AT2 proliferation.

Figure S4:


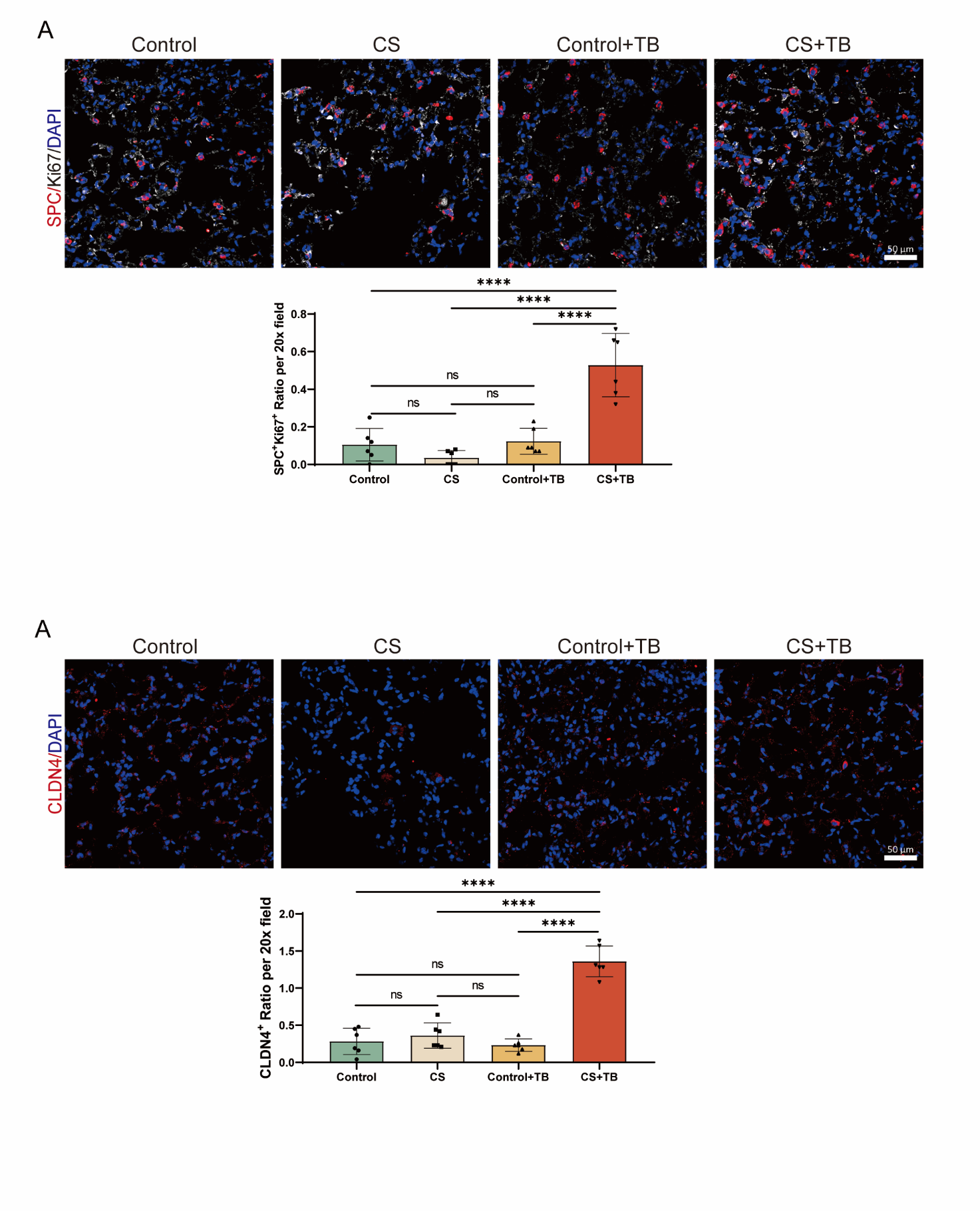


**Fig. S4.** **(A)** Immunofluorescence staining of mouse lung tissues showing SPC (AT2 marker, red), Ki67 (white) and DAPI (blue) in Control, CS, Control+TB, CS+TB groups. Quantification of SPC^+^/Ki67^+^ ratios indicates that TB treatment promotes AT2 proliferation.

Figure S5:


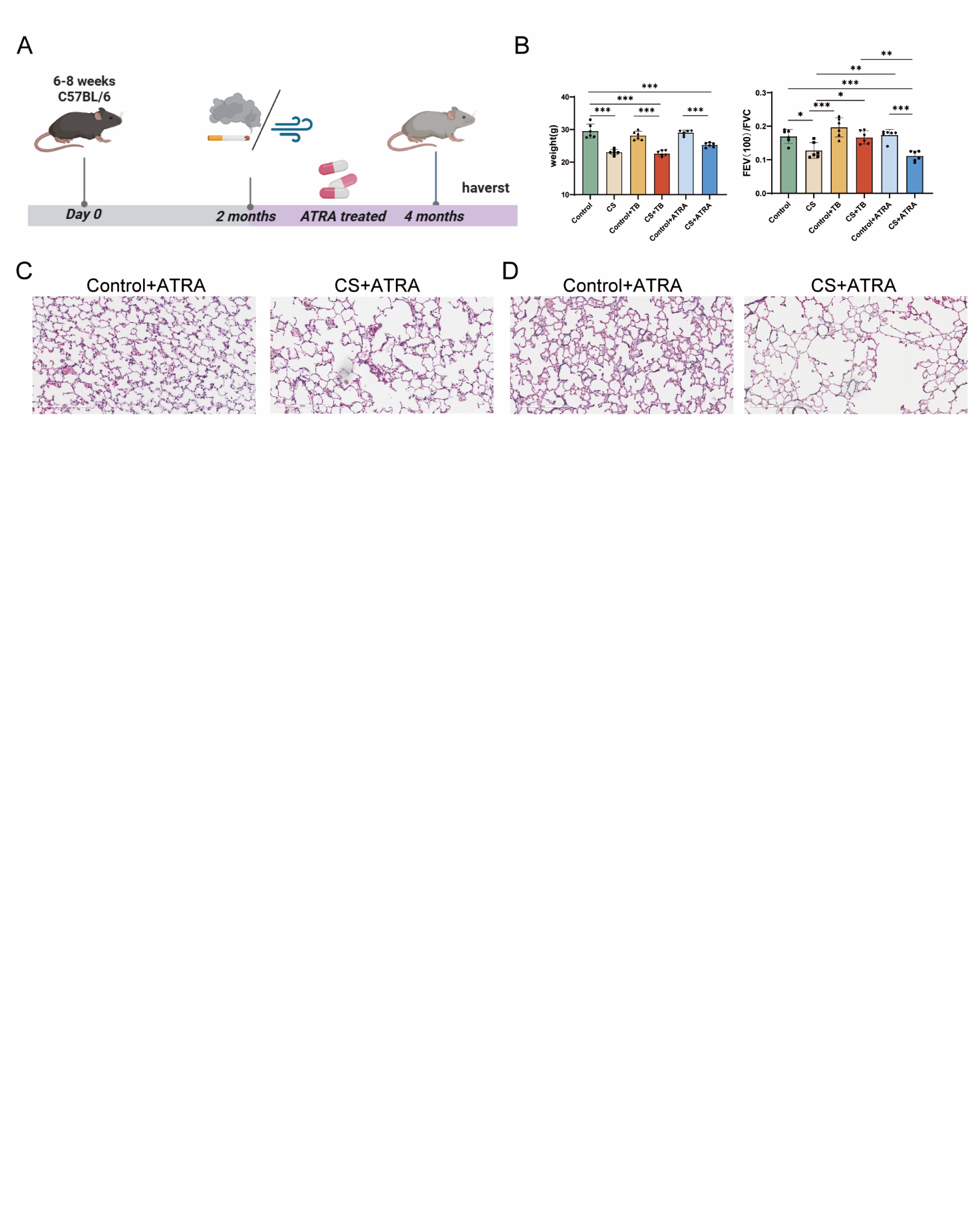


**Fig. S5.** **(A)** Schematic overview of the experimental timeline. **(B)** Body weight and FEV100/FVC ratio measurements in Control, CS, Control + TB, CS + TB, Control + ATRA, and CS + ATRA. Under the present experimental conditions, the CS + TB group showed greater improvement than the CS + ATRA group, particularly in pulmonary function (FEV100/FVC). **(C)** Representative H&E staining of lung tissues. (**D**) Elastin fiber staining of lung tissue.
